# EpCAM Knockdown Alters MicroRNA Expression in Retinoblastoma- Functional Implication of EpCAM Regulated MiRNA in Tumor Progression

**DOI:** 10.1371/journal.pone.0114800

**Published:** 2014-12-12

**Authors:** Madhu Beta, Vikas Khetan, Nivedita Chatterjee, Ganesan Suganeswari, Pukhraj Rishi, Jyotirmay Biswas, Subramanian Krishnakumar

**Affiliations:** 1 L & T Ocular Pathology Department, Kamalanayan Bajaj Research Institute, Vision Research Foundation, No 18/41, College Road, Chennai- 600006, Tamil Nadu, India; 2 Shri Bhagwan Mahavir Vitreoretinal Services and Ocular Oncology Services, Medical Research Foundation, No 18/41, College Road, Chennai-600 006, Tamil Nadu, India; UCSF/VA Medical Center, United States of America

## Abstract

The co-ordinated regulation of oncogenes along with miRNAs play crucial role in carcinogenesis. In retinoblastoma (RB), several miRNAs are known to be differentially expressed. Epithelial cell adhesion molecule (EpCAM) gene is involved in many epithelial cancers including, retinoblastoma (RB) tumorigenesis. EpCAM silencing effectively reduces the oncogenic miR-17-92 cluster. In order to investigate whether EpCAM has wider effect as an inducer or silencer of miRNAs, we performed a global microRNA expression profile in EpCAM siRNA knockdown Y79 cells. MicroRNA profiling in EpCAM silenced Y79 cells showed seventy-three significantly up regulated and thirty-six down regulated miRNAs. A subset of these miRNAs was also validated in tumors. Functional studies on Y79 and WERI-Rb-1 cells transfected with antagomirs against two miRNAs of miR-181c and miR-130b showed striking changes in tumor cell properties in RB cells. Treatment with anti-miR-181c and miR-130b showed significant decrease in cell viability and cell invasion. Increase in caspase-3 level was noticed in antagomir transfected cell lines indicating the induction of apoptosis. Possible genes altered by EpCAM influenced microRNAs were predicted by bioinformatic tools. Many of these belong to pathways implicated in cancer. The study shows significant influence of EpCAM on global microRNA expression. EpCAM regulated miR-181c and miR-130b may play significant roles in RB progression. EpCAM based targeted therapies may reduce carcinogenesis through several miRNAs and target genes.

## Introduction

Retinoblastoma (RB) is an aggressive eye cancer of infancy and childhood. Several over expressed genes have been reported in retinoblastoma (RB) [1]–[3]. Epithelial cell adhesion molecule (EpCAM) is a type I transmembrane glycoprotein over expressed in RB [3]. Several epithelial cancers show up regulation of this protein and it has been considered as a potential molecule for targeted therapy [4]–[7]. The functional significance of EpCAM gene was earlier reported by gene knockdown studies. The study suggested deregulated pathways through differential gene expression profiles on EpCAM silencing [8].

MicroRNAs (miRNAs) are non-coding single stranded small RNA molecules; typically 18–23 nucleotides in length. MicroRNAs are important biological regulators of genes. They prevent the increase in target mRNA levels in cells to maintain the cell metabolism. MicroRNAs control key cellular processes like proliferation, differentiation and apoptosis. The aberrant expression of miRNAs have been identified in various pathologies such as neurodegeneration [9], cardiovascular [10], pulmonary [11], and various cancers [12]. Silencing of EpCAM gene by RNA interference significantly altered the expression of oncogenic microRNA 17–92 cluster [13]. Over expression of miR-17-92 cluster was reported in RB tumours and importance of these miRNAs in RB tumorigenesis was studied through antagomir transfection in Y79 RB cells by our group [13]. Similar to RB, the potential oncogenic nature and over expression of the polycistronic miR-17-92 cluster was reported in other cancers [14], [15]. The tumor suppressor role of miR-34a [16], miR-22 [17], miR-449a/b [18] have also been implicated in RB. In this study we investigated the global microRNA expression affected by EpCAM gene in RB.

We report here that EpCAM silencing resulted in up regulation of 15 miRNA families and down regulates the expression of 25 miRNA families in RB. In addition, miR-181c and miR-130b were thoroughly studied in RB cell lines, on knockdown of EpCAM. Antagomirs against these families lead to decrease in the invasive phenotype and increase in apoptosis. In conclusion, miRNAs regulated by EpCAM have shown to have a potential role in RB progression. Targeting EpCAM regulated miRNAs can aid in formulating therapies against RB.

## Materials

### Cell lines

Y79 and WERI-Rb-1 cell lines were purchased from RIKEN cell bank, Japan.

### Cell culture materials

RPMI-1640 medium (Invitrogen, India), Fetal bovine serum (Sigma, India), Antibiotics and antimycotic solution-100× (HiMedia, India), Lipofectamine-2000 transfection reagent (Invitrogen, India), Poly-L-Lysine (PLL, Sigma, India), MTT [(3-(4,5-dimethylthiazol-2-yl)-2,5-diphenyl tetrazolium bromide] (Sigma, India), Human EpCAM siRNA (Hs_TACSTD1_10; catalogue number SI04343416; Forward strand: GGA ACU CAA UGC AUA ACU ATT and the reverse strand: UAG UUA UGC AUU GAG UUC CCT) and scrambled siRNA (1022563, Qiagen, India), antagomirs: miR-181c (426854-00; miRCURY LNA Power Inhibitor, EXIQON, Denmark) and miR-130b (426777-00; miRCURY LNA Power Inhibitor, EXIQON, Denmark).

### RNA extraction and PCR components

Trizol reagent (Invitrogen, India), miRNA oligos (Eurofins MWG Operon, Bangalore, India), SYBR Green small RNA assay kit (MIR Q-100; Invitrogen, India), NCode First Strand cDNA Synthesis Kit (Invitrogen, India).

### Western blot reagents

EpCAM antibody (C-10, Santa Cruz, India), β-actin antibody (Sigma Aldrich, Bangalore, India), SuperSignal West Femto Substrate (Thermo Scientific, India)

### Assay kits

Caspase-3 assay (Roche Diagnostics, India), BioCoat Matrigel invasion assay kit (354481; Becton Dickinson Biosciences, India).

### Instruments

Spectramax-M4 micro plate reader (Molecular Devices, CA, US), Bioanalyzer (2100; Agilent, Palo Alto, CA).

## Methods

### Tissue samples

RB tumors were collected from children diagnosed with RB. Informed written consent was obtained by Medical Research Foundation, Sankara Nethralaya from the parents/guardians of RB patients for the use of tumor samples from enucleated eyeballs. Three adult non-neoplastic retinas were taken from donor cadaveric eyes received at our CU Shah Eye Bank. This project was reviewed and approved by the ethics committee of Vision Research Foundation Institutional Review Board. The committee agreed and confirmed that the study was acceptable and under the general principles of research and in accordance with the Helsinki Declaration (Project Ethics code: 146-C-2009-P).

### Cell culture

RB cell lines, Y79 and WERI-Rb-1 were cultured in RPMI-1640 media containing 10% FBS and 1X-antibiotic and antimycotic solution. Cells were cultured in flasks at 37°C and 5% CO2.

### EpCAM siRNA transfection

Gene silencing of EpCAM expression was performed as described previously using sequence specific siRNA and transfection reagents [13]. Prior to transfection, six well plates were coated with Poly-L-lysine (PLL) to make the RB suspension cells adhere to the bottom of each plate. Briefly, 2×105 cells/well were plated onto PLL coated six well plates. Complete serum rich RPMI-1640 media was added and cells were allowed to grow for 24–72 hr (until they were 40%–60% confluent). siRNA transfection was carried out as earlier described [13].

### RNA extraction from tissues and EpCAM siRNA treated RB cells

Total RNA was extracted from the siRNA treated, untreated RB cells, RB tumor samples (100–300 mg tissue) and non-neoplastic retina (control), using Trizol reagent according to manufacturer's instruction. Each pellet was air dried and dissolved in RNase free water and stored at −80°C until further use. RNA concentration and purity was checked by UV Spectrophotometry.

### MicroRNA expression profiling using microarray

Microarrays were performed in triplicates for Y79 cell line. The cell line RNA was extracted from treated and untreated cells, followed by a quality check using Bioanalyzer. Hybridization was performed for the biological triplicates. The microarray was then carried out as described previously [19].

### Relative microRNA quantification by real-time quantitative and reverse transcriptase PCR

The detection and quantification of mature miRNA was achieved using real-time PCR. The expression level of miRNAs were quantified in triplicates by qRT-PCR using the human SYBR Green small RNA assay kit. The reverse transcription reaction for miRNA-specific cDNA synthesis was carried out with The NCode First Strand cDNA Synthesis Kit. Quantification was carried out using the manufacturer's protocol starting with 10 ng of the total RNA sample. U6b small RNA was used as a control for normalization. The PCR products were detected with an ABI PRISM 7500 sequence detection system and analysed with the ABI PRISM 7500 SDS software version 2.0.1. The cycle threshold value (C_t_) was determined for each miRNA, and the relative amount of each miRNA to U6b small RNA was calculated using the equation – 2^−ΔΔC^_t_, where ΔC_t_ =  (C_t_ test miRNA – C_t_ control miRNA).

### Antagomir transfection in Y79 and WERI-Rb-1 Retinoblastoma cells

Briefly, 6×10^5^ cells/well were seeded in 6 well plates. Cells were allowed to grow until 50–60% confluent in antibiotic free medium. Antagomirs, miR-181c and miR-130b were transfected and incubated for 24 hr. Antagomirs were prepared at a final concentration of 100 pmol using RNA dilution buffer.

### Cell viability assay

MTT assay was performed on antagomir transfected Y79 and WERI-Rb-1 cells. 5×10^3^ cells were seeded in each well of a 96 well plate. Antagomirs of miR-130b and miR-181c were transfected with opti-MEM media with 20 pmol of miRNA. Opti-MEM media was replaced after 4 hrs of incubation with complete RPMI-1640 media. Readings were taken at 560 nm absorbance.

### Flouorometric caspase-3 assay

Apoptosis marker caspase-3 level was measured in antagomir (miR-130b, miR-181c) treated cells by fluorometric caspase-3 assay. Briefly, 2×10^6^ cells were taken and washed with ice cold PBS. Cells were centrifuged at 300×*g* for 5 min. The cells were resuspended in RIPA (Radio immune precipitation assay; 10 mM Tris-Cl (pH 8.0), 1 mM EDTA, 0.1% SDS, 140 mM NaCl and 1 mM PMSF) lysis buffer followed by centrifugation at 12000×*g*. The supernatant was transferred to a 96 well plate pre-coated with antibodies followed by 1 hr of incubation at 37°C. The solution was removed and washed using a wash buffer. Substrate was added and incubated for 2 hrs at room temperature (RT). Fluorescence reading was taken at λ_max_ ex = 420 nm and λ_max_ em = 480 nm using micro plate reader.

### Matrigel invasion assay

The cell invasion assay was conducted with BioCoat Matrigel. The matrigel chambers were incubated at 37°C for 2 hrs. Briefly, 2×10^5^ cells per well in 1 ml of serum free medium (Opti-MEM) were seeded into pre-coated matrigel chambers. As chemo-attractant, 1 ml of complete medium was added to the bottom of each well. To the seeded cells, a prepared complex of 10 µl Lipofectamine-2000 and 100 pmol antagomir was added and incubated for 12 hrs. Serum free media was then replaced with complete serum media. After 48 hrs of transfection, matrigel was swabbed with pre wetted cotton. The lower surface of the chamber was fixed with 0.5 ml of methanol for 2 min. Fixed cells were stained using Crystal Violet and finally rinsed with water and air dried. Several fields of invaded cells were counted under inverted microscope in triplicate experiments. The percentage of invasion was calculated according to the following formulae. 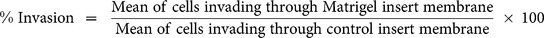



### Western blot analysis

Y79, WERI-Rb-1 and MCF-7 cells were lysed in mammalian cell lysis buffer using a sonicator (22.5 KHz, 3×15 sec) on ice for 15 min. 100 µg of protein was electrophoresed with 12% sodium dodecyl sulfate-polyacrylamide gel and blotted onto nitrocellulose membrane. Membranes were blocked in 5% BSA and then incubated separately with 1∶500 diluted mouse monoclonal primary antibody against EpCAM overnight at 4°C. β-actin was used as a loading control (1∶5000). After washing, the membranes were incubated with horseradish peroxidase-conjugated anti-mouse IgG antibody (1∶2000) for 1 hr at RT. The bands were developed using luminol reagent and images captured in a Chemidoc system.

### Bioinformatics prediction of target genes for miRNA and chromosomal locations

Target genes, their respective gene ontologies (GO terms) and pathways were predicted for all the significant differential miRNAs of Y79 using GeneSpring GX version 11.5 software. A Cytoscape imaging tool was used to draw the microRNA and important target gene interactions for miR-130b and miR-181c [19], [20]. TAM (The tool for annotations of human miRNAs) tool was used for miRNA classification (**File S 1**).

### Statistical analysis

All the Real time data analysis was performed using ABI-7500 software version-2.0.1. Data was normalized according to default parameters. Correlation statistics were checked with Graph pad prism version-6. The microarray raw data files were imported to Gene Spring GX software version 11.5 for log_2_ transformation. Signal cut-off measurements were set to 1.0, and normalized to 90th percentile of signal intensity to standardize each chip for cross-array comparison. Significant differential miRNAs were obtained by using unpaired Student's t test with p-value cut off <0.05.

## Results

### Clinico-pathological information of RB tumors

The clinico-pathological features of RB tumors studied for EpCAM (n = 30) and miRNA (n = 20) provided in **S1 Table**. The mean ±SD of patients included in the study was 3.3±2.1 years and comprised of 18 boys and 16 girls. All the RB tumors were unilateral. Thirteen cases without any invasion of optic nerve or choroid and twenty one cases with invasion of either optic nerve, choroid or both were chosen.

### Quantification of EpCAM by qRT-PCR shows high expression and siRNA knockdown for EpCAM leads to down regulation

**Figure 1:**
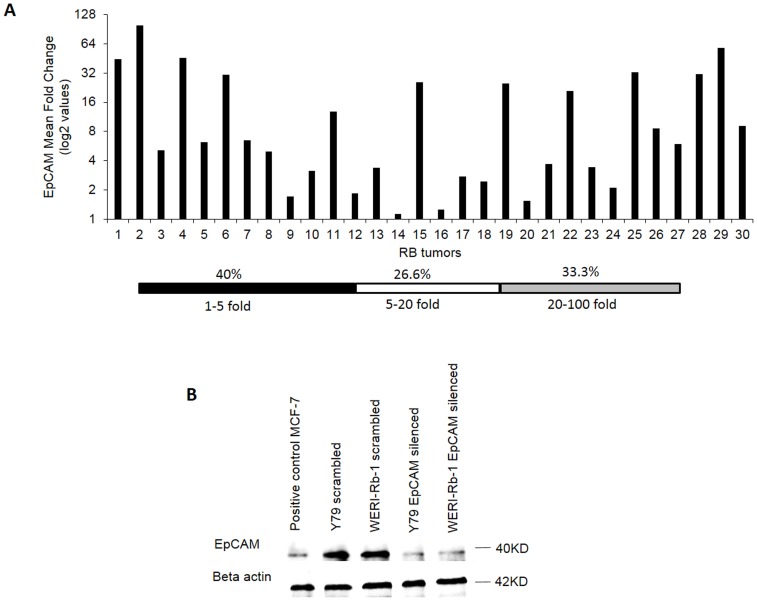
EpCAM is expressed in RB tumors. (A) EpCAM mRNA ranges from 2-100 fold in RB tumors (n = 30). Pooled non-neoplastic retinas (n = 3) was used as control. Data is expressed as fold change relative to control. (B) Immunoblot for protein levels of EpCAM siRNA knockdown in Y79 and WERI-Rb-1 cell lines.

All the 30 RB tumors showed EpCAM mRNA expression in qRT-PCR validation (**Fig. 1A** **,** **S1 Table**). In our study 60% RB tumors (n = 30) showed more than 5 fold expression of EpCAM. EpCAM protein levels decreased in both Y79 and WERI-Rb-1 cells on silencing with EpCAM siRNA. MCF-7 cells were used as positive control showed EpCAM expression (**Fig. 1B**).

### Microarray analysis revealed differential expression of miRNAs in EpCAM silenced Y79 cells

For miRNA microarray data, differential miRNAs was filtered using two criteria; (1) a log2 fold change geo mean cut off level of> = 0.8 for up regulated and a log2 fold change geo mean cut off of < = 0.8 for down regulated miRNAs [21], and (2) a significant p-value (<0.05) derived from student's t-test. Based on the above screening, we obtained 73 up regulated miRNAs and 36 down regulated miRNAs in Y79 EpCAM knockdown cells (**S1** and **S2 File**). MicroRNA classification identified more number of down regulated families than up regulated ones. Significant among the up regulated families were miR-154, and miR-30. The most significant down regulated families were miR-17, -181, -15, -320 and Let-7 family. We have chosen two miR families which were down regulated in post-EpCAM knockdown and therefore likely to be oncogenic.

### EpCAM silencing significantly decreased the expression of miR-130 and miR-181 family members in RB cell lines

**Figure 2:**
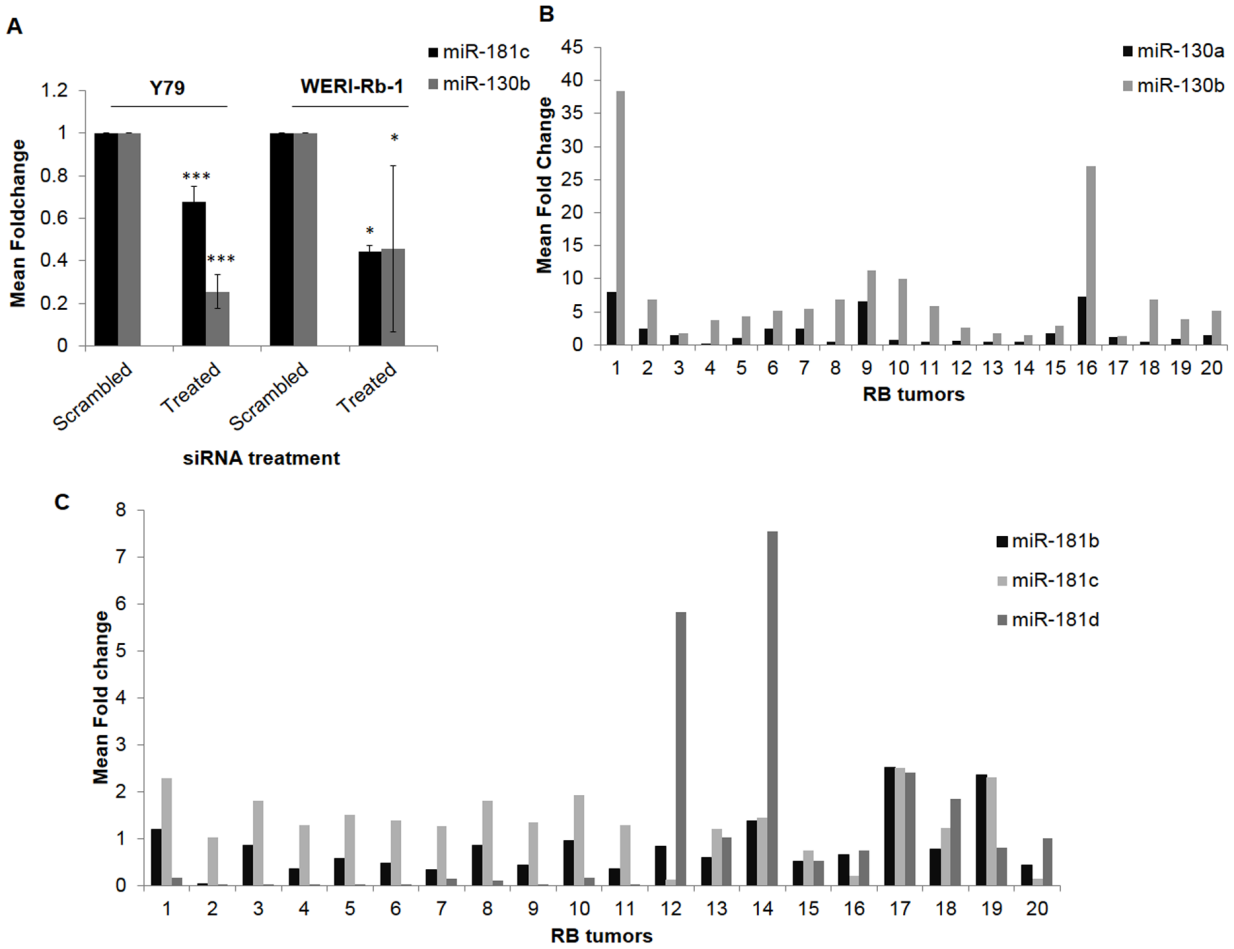
Quantitative Real Time PCR validation confirmed expression of miR-130b and miR-181c in RB tumors and microarray results in EpCAM silenced Y79 cells. A) miR-130b and miR-181c were quantified in Y79 and WERI-Rb-1 after EpCAM siRNA. Values are represented as mean ±SD in three independent experiments (*p<0.05). B) miR-130a, 130b expression ranged from 1–35 folds in the RB tumors. C) miR-181b, 181c, 181d, 130a, and 130b were quantified in twenty RB tumors. MiR-181b, miR-181c and miR-181d expression ranged from 1–7 fold.

Microarray experiments showed that miR-130 and miR-181 families reduced significantly with log_2_ fold change of −0.8 for miR- 181b, −5.03 for miR-181c, −0.57 for miR-181d, −0.63 for miR-130a, −0.63 for miR-130b (p<0.01*** for all miRNAs) (**S1** and S2 **File**). Real time PCR validation in cell line obtained a mean fold change of −0.6 for miR-181c and −0.2 for miR-130b (p<0.05*) in Y79. In WERI-Rb-1 cells, the fold changes were −0.4 for miR-181c and −0.4 for miR-130b (**Fig. 2A**).

### Primary RB tumors showed high expression of miR-130b family and miR-181 family members

The importance of miR-181 and miR-130 families were further tested in 20 tumors. Quantitative real time PCR method was used to find the expression of miR-130 and miR-181 family members. The mean fold changes of miRNAs were found to be 0.8±1.2 for miR-181b, 1.3±0.6 for miR-181c, 1.1±2.0 for miR-181d (**Fig. 2B**), 2.0±2.3 for miR-130a and 7.6±9.1 for miR-130b (**Fig. 2C**), respectively.

### Functional role of miR-130b and miR-181c in retinoblastoma cell line in proliferation, invasion and apoptosis

**Figure 3:**
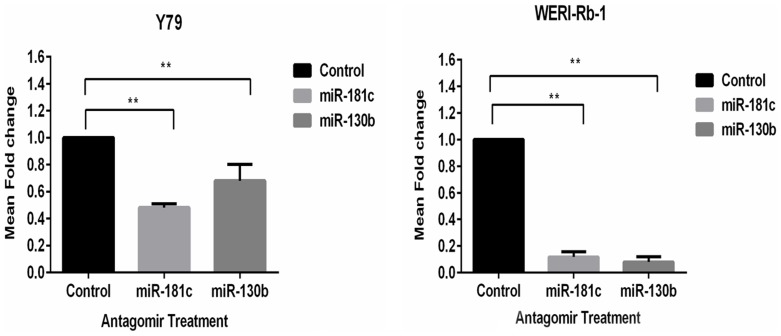
Antagomir treatment in Y79 and WERI-Rb-1 cell line lead to suppression of miR-130b and miR-181c. Decrease in miR-130b and miR-181c levels occur in antagomir treated Y79 and WERI-Rb-1 cell lines (*p<0.05). Quantification was done by qRT-PCR.

The functional importance of the tumor significant miRNAs was dissected by performing cell based assays on inhibiting miR-181c and miR-130b. Standardisation with miR specific antagomirs showed significant decrease in miR-130b and miR-181c in treated Y79 and WERI-Rb-1 cells (p<0.05**). Down regulation of miR-130b, miR-181c was −0.4 and −0.6 fold in Y79 cells and −0.1 and −0.01 fold in WERI-Rb-1 cells (**Fig. 3**).

#### a) Antagomirs of miR-130b and miR-181c decreased cell viability in Y79 and WERI-Rb-1

**Figure 4:**
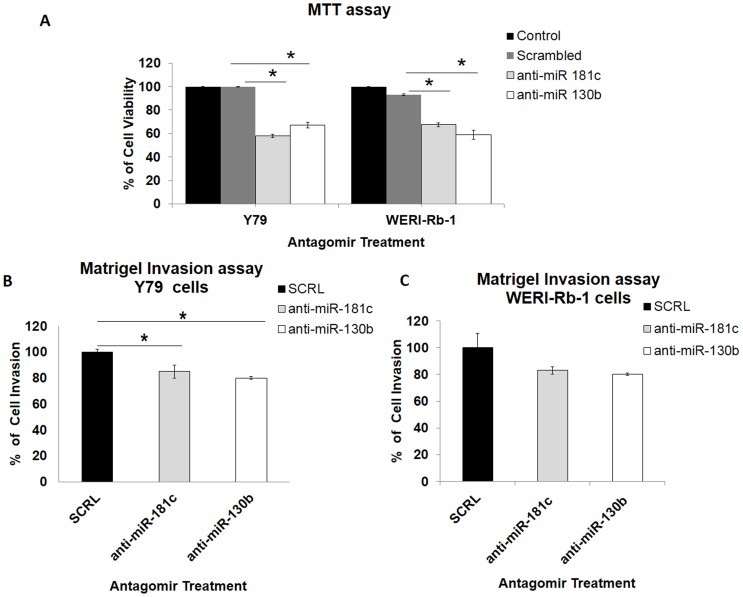
Inhibition of miR-181c and miR-130b decreased cell viability and invasion in Y79 and WERI-Rb-1 cells. A) Percentage of cell viability changes in anti-miR181c and anti-miR130b transfected Y79 and WERI-Rb-1 cells. Significant decrease in cell viability was noticed in both cell lines (*p<0.05). MTT was used for assessing cell viability. B) Decrease in cell invasion by 20% is observed for anti-miR-130b and 12% for anti-miR-181c transfected Y79 cells. C) Decrease in cell invasion by 17% on treatment with anti-miR-130b and 20% in cell viability with anti-miR-181c is seen in transfected WERI-Rb-1 cells. Lack of significant p value reiterates non-invasive property of this cell lines. Data shown represent mean ±SD from three independent experiments.

Antagomirs were used for the inhibition of miR-130b and miR-181c. Cell proliferation decreased significantly; 41.9% (Y79 cells) for miR-181c and 32.8% (Y79 cells) for miR-130b antagomir treated cells (p<0.05*). In treated WERI-Rb-1, decrease in cell proliferation by 32.5% for miR-181c and 41.1% for miR-130b (p<0.05*) were seen (**Fig. 4A**).

#### b) Cell invasion reduced in antagomir (miR-181c and miR-130b) transfected Y79 and WERI-Rb-1 cells

Cell invasion assay in Y79 cells show that on inhibiting miR-130b and miR-181c, migration decreases by 20% and 15%, respectively (p<0.05*) (**Fig. 4B**, **S3 File**). Percentage decrease was 17% for miR-181c and 20% for miR-130b in WERI-Rb-1 cells (p<0.05*) (**Fig. 4C**, **S3 File**).

#### c) Increase in caspase-3 level in antagomir (miR-181c, miR-130b) treated Y79 and WERI-Rb-1 cells suggest activation of cell death pathways

**Figure 5:**
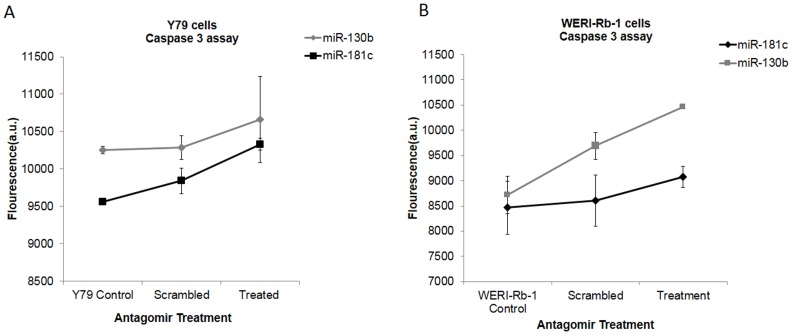
Increase in caspase-3 level is observed in antagomir treated Y79 and WERI-Rb-1 cells. Increase in caspase-3 level occurs in A) Y79 and B) WER-Rb-1 transfected with anti-miR-181c and anti-miR-130b. Caspase-3 was measured by fluorometric assay. Control cells were untreated. Values represented in the form of mean ±SD are from three independent experiments.

Apoptosis was measured by investigating level of caspase-3 protein. Increase in fluorescence units (au) of caspase-3 in miR-181c, or miR-130b antagomir treated Y79 and WERI-Rb-1 compared to mock miRNA treated cells suggested the involvement of apoptotic cell death pathways (**Fig. 5A, B** **)**. U937 cells treated with camptothecin were used as positive control cells (data not shown).

### Correlation of EpCAM expression and miR-130b, miR-181c in primary RB tumors

To investigate whether a correlation in expression indeed exists between the miR studied and EpCAM in RB, we performed correlation analysis. However, there was no positive correlation observed between EpCAM and miR-130b, miR-181c members.

### *In silico* chromosomal mapping for differential microRNA of EpCAM

Significant microRNAs mapping to different chromosomal regions, show that among the miRNA that were down regulated distribution was limited to; 20% on ChrX (miR-362, miR-532, miR-500*, miR-500, miR-501*, miR-532* & miR-98), 12.5% on Chr9, 10% on Chr13, and 7.5% on Chr1 and 7 (**S4 File**). Up regulated miRNA had similar localised distribution; 9.3% on Chr19 (miR-150*, miR-125a*, miR-520b, miR-371), 9.3% on Chr14 (miR-127*, miR-382, miR-485, miR-300, miR-494, miR-134), 8% on Chr1, 6.6% on Chr16 as well as 6, 5.3% ChrX and Chr4 (**S5 File**).

## Discussion

High expression of EpCAM supports tumor progression in RB [3], [8]. In depth studies demonstrated that EpCAM acts as a potent signal transducer that uses components of the Wnt pathway, with an active involvement in cell proliferation [22], [23]. We postulated that EpCAM may influence multiple microRNA clusters/families in RB.

In the current study, silencing EpCAM in Y79 cells showed 109 significantly differentially expressed miRNAs in microarray profiling. This includes our earlier reported miR-17-92 cluster [13]. Further classification of these miRNAs identified miR-181 [24], [25], miR-17 [26], miR-320 [27], miR-130 and Let-7 [28] as significantly down regulated families. miR-15, miR-23, and miR-27 though not reported in RB have some of their members associated in other cancers [29].

We selected two microRNA families, miR-181(miR-181b, miR-181c and miR-181d) and miR-130 (miR-130a and miR-130b) families based on their previous association with EpCAM and literature reports of cancer to find out their role in RB tumor cell proliferation. Previous studies on miR-181 family in hepatocellular carcinoma showed a regulatory link between miR-181 family and EpCAM positive cancer cells [24], [25]. The oncogenic potential and over expression of miR-130b was reported in multiple cancers; colorectal [30], gastric [31], [32], and renal carcinoma [33]. High expression and the oncogenic role of miR-130a is also observed in colorectal [34] and ovarian cancers [35]. In a cohort of twenty tumors, we consistently observed high expression of miR-181 family members and miR-130b family. Significantly expressed miR-181c and miR-130b (p<0.05) were taken for antagomir studies to investigate their functional role associated with RB.

In vitro functional studies; cell viability, apoptosis and cell invasion study were performed using antagomirs of miR-130b and miR-181c in Y79 and WERI-Rb-1 cells. Cell viability assay shows that viability was decreased significantly in both Y79 and WERI-Rb-1. The decrease of cell viability for anti-miR-130b is less in Y79 compared to anti-miR-181c in Y79 cells. In contrast decrease in cell viability is more for anti-miR-130b compared to anti-miR-181c treatment in WERI-Rb-1 cells. To support this, we analysed caspase-3 cascade in Y79 and WERI-Rb-1 cells. Increase in fluorescence of caspase-3 in both miR-181c, and miR-130b antagomir treated Y79 and WERI-Rb-1 cells confirmed the role of these miRNAs in cell apoptosis. Subsequently, the inhibitory effect of these antagomirs on cell invasion was studied using Matrigel chambers. We observed a significant decrease in cell invasion in antagomir treated Y79 cells but not noticeably in WERI-Rb-1 cells. It may be noted that WERI-Rb-1 cells are known to be less invasive [36].

Gene ontologies were predicted for miR-181c and miR-130b targeted genes. We found that many genes were implicated in Wnt signalling and other important pathways which play a major role in tumorigenesis. We sought to investigate with bio-informatic tools whether differentially expressed miRNAs of EpCAM have any association with chromosomal aberrations. *In silico* chromosomal mapping was performed for differentially regulated miRNAs in EpCAM silenced Y79 data. We addressed the following queries based on the chromosomal locations of EpCAM regulated miRNAs; 1) The relationship between site fragility and miRNA density/miRNA distribution on the chromosomes, 2) The locus of EpCAM gene versus the loci of miRNAs. It was observed that many miRNA were associated with ChrX, Chr9 and Chr13. Frequent chromosomal aberrations in RB were reported for ChrX and Chr13 [37], [38], miR-181c which was up regulated in RB tumors is associated with 19p13 chromosomal gain region of RB [3], [39]. Among other significantly changing families, miR-101 and miR-30e are associated with Chr1p gain region [37]. Several of these play important functions in cancer [40] and immune disorders [41]. The complete set of miR-362, miR-532, miR-500*, miR-500, miR-501*, miR-532* and miR-98 located on ChrX had been reported with chromosomal gain region in B-cell lymphoma [42]. Unusually, miRNA which in our experimental data show up regulation on silencing EpCAM, are theoretically expected to be down regulated in tumors, since they are tumor suppressors. All of these are located in chromosomal gain regions in our bioinformatics analysis. This suggests that EpCAM mediates the control of these miRNA through multiple target genes and other protein interactions.

In conclusion, EpCAM a potential oncogene is a master regulator of several miRNAs and genes which are necessary for RB tumor progression. Existing literature has implicated many of these miRNA regulated by EpCAM in various types of cancers; it is likely that these miRNAs have a strong role in common cancer pathways. The miRNAs regulated by EpCAM control oncogenic, tumor suppressive and also metabolic functions. MiR-130b and miR-181c that we studied here affected RB cell proliferation, invasion and apoptosis. MicroRNAs can regulate multiple pathways in cancer through a complex and intricate network of gene interactions. It has also been suggested that they can be good therapeutic targets [43]. However, the large number of families affected as evidenced in this study and their very interactive nature makes them difficult candidates for therapy. It may be more worthwhile to target a potent cancer specific gene like EpCAM that controls several miRNA for RB tumor progression.

## Supporting Information

S1 Table **Clinical profile of RB tumors with fold change values of EpCAM, miR-181c and miR-130b.** Table showing clinico-pathological information of retinoblastoma tumors and fold change values obtained by qRT-PCR for EpCAM, miR-130b and miR-181c.
(XLSX)

S1 File **Microarray identified post EpCAM silenced miRNAs and Gene Targets.** Differential miRNAs with significant p values(<0.05) are given. Gene targets, Gene ontologies and differential miRNA classification are given in the table.
(XLS)

S2 File **Effect of EpCAM gene knockdown on miRNA expression profile in Y79 cells.** MicroRNA expression profile in Y79 cells determined by microarray. Silencing of EpCAM lead to differentially expressed miRNAs. Heat map shows hierarchical arrangement based on fold change in Y79/EpCAM siRNA and Y79/Control. Green denotes low expression level and red denotes high expression level.
(TIF)

S3 File **Representative images of invasion assay.** Cells invading into matrigel were fixed, stained with Crystal Violet and photographed in 10× magnification field. Invaded cells are indicated by black arrows in Y79 and WERI-Rb-1 cell controls. Control, scrambled and treated chambers of Y79 and WERI-Rb-1 are shown.
(TIF)

S4 File ***In silico*** **representation of EpCAM downregulated miRNA on chromosomal regions.** Chromosomal locations of significant down regulated miRNAs upon EpCAM silencing in Y79 cells. EpCAM is mapped to p-arm of Chromosome-2 (blue dot). miRNAs are labelled as lines on the 24 chromosomes. Polycistronic microRNAs-miR-17, miR-18a, miR-20a, miR-19b located on 13q31.3, miR-10, miR-30e located on chromosome-1 are associated with RB chromosomal gain regions. miRNAs (non-polycistronic), miR-362, miR-532, miR-500*, miR-500, miR-501*, miR-532* & miR-98 were located at Chromosomal-Xp11.
(TIF)

S5 File ***In silico*** **representation of significantly up regulated miRNAs on EpCAM silencing in chromosomal regions.** Details of chromosomal locations of significant miRNAs up regulated upon EpCAM silencing in Y79 cells. EpCAM is mapped to p-arm of Chromosome-2 (blue dot). miRNAs are labelled as lines on the 24 chromosomes. miR-127-3p, miR-382, miR-485, miR-300, miR-494, miR-134 map to chromosomal-14q32 region and miR-150*, miR-125a-3p, miR-520b, miR-371 map to chromosome-19q13.4 regions.
(TIF)

## References

[1] Zhang J, Benavente CA, McEvoy J, Flores-Otero J, Ding L, et al (2012) A novel retinoblastoma therapy from genomic and epigenetic analyses. Nature 481:329–334. 22237022 10.1038/nature10733 PMC3289956

[2] Madhavan J, Coral K, Mallikarjuna K, Corson TW, Amit N, et al (2007) High expression of KIF14 in retinoblastoma: association with older age at diagnosis. Invest Ophthalmol Vis Sci 48:4901–4906. 17962437 10.1167/iovs.07-0063

[3] Krishnakumar S, Mohan A, Mallikarjuna K, Venkatesan N, Biswas J, et al (2004) EpCAM expression in retinoblastoma: a novel molecular target for therapy. Invest Ophthalmol Vis Sci 45:4247–4250. 15557427 10.1167/iovs.04-0591

[4] Matsuda T, Takeuchi H, Matsuda S, Hiraiwa K, Miyasho T, et al (2014) EpCAM, a Potential Therapeutic Target for Esophageal Squamous Cell Carcinoma. Ann Surg Oncol 21 Suppl 3: 356–364. 24566863 10.1245/s10434-014-3579-8

[5] Bellone S, Siegel ER, Cocco E, Cargnelutti M, Silasi DA, et al (2009) Overexpression of epithelial cell adhesion molecule in primary, metastatic, and recurrent/chemotherapy-resistant epithelial ovarian cancer: implications for epithelial cell adhesion molecule-specific immunotherapy. Int J Gynecol Cancer 19:860–866. 19574774 10.1111/IGC.0b013e3181a8331f

[6] Brunner A, Schaefer G, Veits L, Brunner B, Prelog M, et al (2008) EpCAM overexpression is associated with high-grade urothelial carcinoma in the renal pelvis. Anticancer Res 28:125–128. 18383834

[7] Sithambaram D, Palanivelu S, Subramanian K, Sahoo S, Verma RS (2011) Specific targeting of Ep-CAM in various carcinomas by novel monoclonal antibodies. Hybridoma (Larchmt) 30:511–518. 22149275 10.1089/hyb.2011.0069

[8] Mitra M, Kandalam M, Verma RS, UmaMaheswari K, Krishnakumar S (2010) Genome-wide changes accompanying the knockdown of Ep-CAM in retinoblastoma. Mol Vis 16:828–842. 20461151 PMC2866575

[9] Schaefer A, O'Carroll D, Tan CL, Hillman D, Sugimori M, et al (2007) Cerebellar neurodegeneration in the absence of microRNAs. J Exp Med 204:1553–1558. 17606634 10.1084/jem.20070823 PMC2118654

[10] Thum T, Gross C, Fiedler J, Fischer T, Kissler S, et al (2008) MicroRNA-21 contributes to myocardial disease by stimulating MAP kinase signalling in fibroblasts. Nature 456:980–984. 19043405 10.1038/nature07511

[11] Hassan T, McKiernan PJ, McElvaney NG, Cryan SA, Greene CM (2012) Therapeutic modulation of miRNA for the treatment of proinflammatory lung diseases. Expert Rev Anti Infect Ther 10:359–368. 22397568 10.1586/eri.11.175

[12] Wiemer EA (2007) The role of microRNAs in cancer: no small matter. Eur J Cancer 43:1529–1544. 17531469 10.1016/j.ejca.2007.04.002

[13] Kandalam MM, Beta M, Maheswari UK, Swaminathan S, Krishnakumar S (2012) Oncogenic microRNA 17–92 cluster is regulated by epithelial cell adhesion molecule and could be a potential therapeutic target in retinoblastoma. Mol Vis 18:2279–2287. 22969266 PMC3436882

[14] Hayashita Y, Osada H, Tatematsu Y, Yamada H, Yanagisawa K, et al (2005) A polycistronic microRNA cluster, miR-17-92, is overexpressed in human lung cancers and enhances cell proliferation. Cancer Res 65:9628–9632. 16266980 10.1158/0008-5472.CAN-05-2352

[15] Mogilyansky E, Rigoutsos I (2013) The miR-17/92 cluster: a comprehensive update on its genomics, genetics, functions and increasingly important and numerous roles in health and disease. Cell Death Differ 20:1603–1614. 24212931 10.1038/cdd.2013.125 PMC3824591

[16] Dalgard CL, Gonzalez M, deNiro JE, O'Brien JM (2009) Differential microRNA-34a expression and tumor suppressor function in retinoblastoma cells. Invest Ophthalmol Vis Sci 50:4542–4551. 19443717 10.1167/iovs.09-3520

[17] Sreenivasan S, Thirumalai K, Danda R, Krishnakumar S (2012) Effect of curcumin on miRNA expression in human Y79 retinoblastoma cells. Curr Eye Res 37:421–428. 22510010 10.3109/02713683.2011.647224

[18] Martin A, Jones A, Bryar PJ, Mets M, Weinstein J, et al (2013) MicroRNAs-449a and -449b exhibit tumor suppressive effects in retinoblastoma. Biochem Biophys Res Commun 440:599–603. 24120948 10.1016/j.bbrc.2013.09.117

[19] Beta M, Venkatesan N, Vasudevan M, Vetrivel U, Khetan V, et al (2013) Identification and Insilico Analysis of Retinoblastoma Serum microRNA Profile and Gene Targets Towards Prediction of Novel Serum Biomarkers. Bioinform Biol Insights 7:21–34. 23400111 10.4137/BBI.S10501 PMC3547501

[20] Delfino KR, Rodriguez-Zas SL (2013) Transcription factor-microRNA-target gene networks associated with ovarian cancer survival and recurrence. PLoS One 8:e58608. 23554906 10.1371/journal.pone.0058608 PMC3595291

[21] Lagatie O, Van Loy T, Tritsmans L, Stuyver LJ (2014) Circulating human microRNAs are not linked to JC polyomavirus serology or urinary viral load in healthy subjects. Virol J 11:41. 24588811 10.1186/1743-422X-11-41 PMC3945012

[22] Yamashita T, Budhu A, Forgues M, Wang XW (2007) Activation of hepatic stem cell marker EpCAM by Wnt-beta-catenin signaling in hepatocellular carcinoma. Cancer Res 67:10831–10839. 18006828 10.1158/0008-5472.CAN-07-0908

[23] Maetzel D, Denzel S, Mack B, Canis M, Went P, et al (2009) Nuclear signalling by tumour-associated antigen EpCAM. Nat Cell Biol 11:162–171. 19136966 10.1038/ncb1824

[24] Ji J, Yamashita T, Budhu A, Forgues M, Jia HL, et al (2009) Identification of microRNA-181 by genome-wide screening as a critical player in EpCAM-positive hepatic cancer stem cells. Hepatology 50:472–480. 19585654 10.1002/hep.22989 PMC2721019

[25] Ji J, Yamashita T, Wang XW (2011) Wnt/beta-catenin signaling activates microRNA-181 expression in hepatocellular carcinoma. Cell Biosci 1:4. 21711587 10.1186/2045-3701-1-4 PMC3116242

[26] Conkrite K, Sundby M, Mukai S, Thomson JM, Mu D, et al (2011) miR-17∼92 cooperates with RB pathway mutations to promote retinoblastoma. Genes Dev 25:1734–1745. 21816922 10.1101/gad.17027411 PMC3165937

[27] Zhao JJ, Yang J, Lin J, Yao N, Zhu Y, et al (2009) Identification of miRNAs associated with tumorigenesis of retinoblastoma by miRNA microarray analysis. Childs Nerv Syst 25:13–20. 18818933 10.1007/s00381-008-0701-x

[28] Mu G, Liu H, Zhou F, Xu X, Jiang H, et al (2010) Correlation of overexpression of HMGA1 and HMGA2 with poor tumor differentiation, invasion, and proliferation associated with let-7 down-regulation in retinoblastomas. Hum Pathol 41:493–502. 20004941 10.1016/j.humpath.2009.08.022

[29] Theriault BL, Dimaras H, Gallie BL, Corson TW (2014) The genomic landscape of retinoblastoma: a review. Clin Experiment Ophthalmol 42:33–52. 24433356 10.1111/ceo.12132 PMC3896868

[30] Colangelo T, Fucci A, Votino C, Sabatino L, Pancione M, et al (2013) MicroRNA-130b Promotes Tumor Development and Is Associated with Poor Prognosis in Colorectal Cancer. Neoplasia 15:1218–1231. 24027433 10.1593/neo.13998 PMC3769887

[31] Lai KW, Koh KX, Loh M, Tada K, Subramaniam MM, et al (2010) MicroRNA-130b regulates the tumour suppressor RUNX3 in gastric cancer. Eur J Cancer 46:1456–1463. 20176475 10.1016/j.ejca.2010.01.036

[32] Kim BH, Hong SW, Kim A, Choi SH, Yoon SO (2013) Prognostic implications for high expression of oncogenic microRNAs in advanced gastric carcinoma. J Surg Oncol 107:505–510. 22996433 10.1002/jso.23271

[33] Wu X, Weng L, Li X, Guo C, Pal SK, et al (2012) Identification of a 4-microRNA signature for clear cell renal cell carcinoma metastasis and prognosis. PLoS One 7:e35661. 22623952 10.1371/journal.pone.0035661 PMC3356334

[34] Liu L, Nie J, Chen L, Dong G, Du X, et al (2013) The oncogenic role of microRNA-130a/301a/454 in human colorectal cancer via targeting Smad4 expression. PLoS One 8:e55532. 23393589 10.1371/journal.pone.0055532 PMC3564758

[35] Yang L, Li N, Wang H, Jia X, Wang X, et al (2012) Altered microRNA expression in cisplatin-resistant ovarian cancer cells and upregulation of miR-130a associated with MDR1/P-glycoprotein-mediated drug resistance. Oncol Rep 28:592–600. 22614869 10.3892/or.2012.1823

[36] Chevez-Barrios P, Hurwitz MY, Louie K, Marcus KT, Holcombe VN, et al (2000) Metastatic and nonmetastatic models of retinoblastoma. Am J Pathol 157:1405–1412. 11021842 10.1016/S0002-9440(10)64653-6 PMC1850157

[37] Van der Wal JE, Hermsen MA, Gille HJ, Schouten-Van Meeteren NY, Moll AC, et al (2003) Comparative genomic hybridisation divides retinoblastomas into a high and a low level chromosomal instability group. J Clin Pathol 56:26–30. 12499428 10.1136/jcp.56.1.26 PMC1769844

[38] Serena-Lungarotti M, Calabro A, Mariotti G, Mastroiacovo PP, Provenzano S, et al (1979) Interstitial deletion 13q syndromes: a report on two unrelated patients. Hum Genet 52:269–274. 535887 10.1007/BF00278676

[39] Rushlow DE, Mol BM, Kennett JY, Yee S, Pajovic S, et al (2013) Characterisation of retinoblastomas without RB1 mutations: genomic, gene expression, and clinical studies. Lancet Oncol 14:327–334. 23498719 10.1016/S1470-2045(13)70045-7

[40] Singhal R, Bard JE, Nowak NJ, Buck MJ, Kandel ES (2013) FOXO1 regulates expression of a microRNA cluster on X chromosome. Aging (Albany NY) 5:347–356. 23748164 10.18632/aging.100558 PMC3701110

[41] Pinheiro I, Dejager L, Libert C (2011) X-chromosome-located microRNAs in immunity: might they explain male/female differences? The X chromosome-genomic context may affect X-located miRNAs and downstream signaling, thereby contributing to the enhanced immune response of females. Bioessays 33:791–802. 21953569 10.1002/bies.201100047

[42] Li C, Kim SW, Rai D, Bolla AR, Adhvaryu S, et al (2009) Copy number abnormalities, MYC activity, and the genetic fingerprint of normal B cells mechanistically define the microRNA profile of diffuse large B-cell lymphoma. Blood 113:6681–6690. 19278952 10.1182/blood-2009-01-202028 PMC3401058

[43] Bader AG, Brown D, Stoudemire J, Lammers P (2011) Developing therapeutic microRNAs for cancer. Gene Ther 18:1121–1126. 21633392 10.1038/gt.2011.79 PMC3910432

