# Correction: EpCAM Knockdown Alters MicroRNA Expression in Retinoblastoma- Functional Implication of EpCAM Regulated MiRNA in Tumor Progression

**DOI:** 10.1371/journal.pone.0119745

**Published:** 2015-04-10

**Authors:** The PLOS ONE Staff

There is a missing affiliation for Dr. Madhu Beta. Madhu Beta is affiliated with 1. L & T Ocular Pathology Department, Kamalanayan Bajaj Research Institute, Vision Research Foundation, No 18/41, College Road, Chennai- 600006, Tamil Nadu, India and 3. CeNTAB, SASTRA (Shanmugha Arts, Science,Technology & Research Academy) University, Thanjavur, India.
