# Supplementary figures and images for: EpCAM Knockdown Alters MicroRNA Expression in Retinoblastoma- Functional Implication of EpCAM Regulated MiRNA in Tumor Progression
Source: PLoS One. 2014 Dec 12;9(12):e114800. doi: 10.1371/journal.pone.0114800 (PMC4264963; doi:10.1371/journal.pone.0114800)

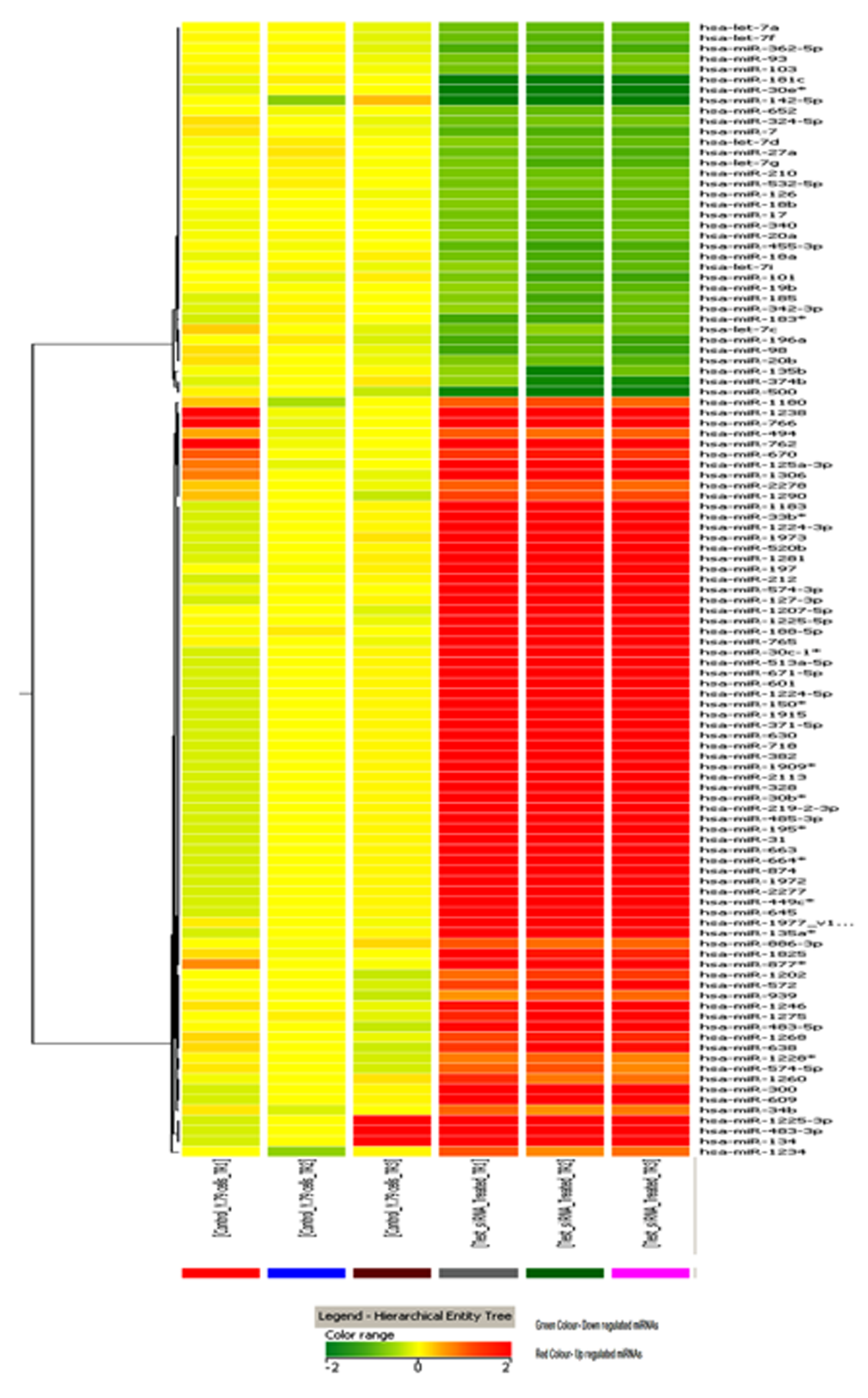

Supplement: S2 File — Effect of EpCAM gene knockdown on miRNA expression profile in Y79 cells. MicroRNA expression profile in Y79 cells determined by microarray. Silencing of EpCAM lead to differentially expressed miRNAs. Heat map shows hierarchical arrangement based on fold change in Y79/EpCAM siRNA and Y79/Control. Green denotes low expression level and red denotes high expression level. (TIF) [file pone.0114800.s003.tif]

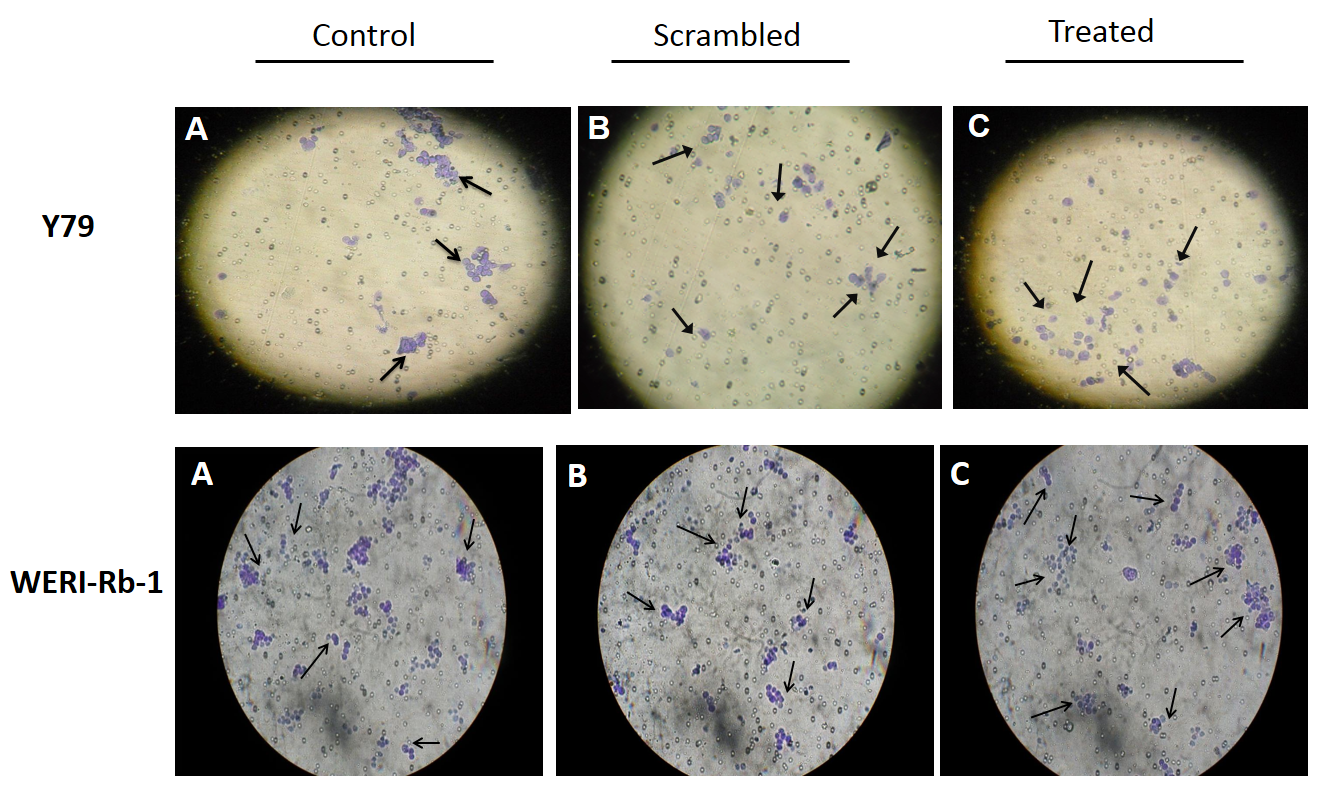

Supplement: S3 File — Representative images of invasion assay. Cells invading into matrigel were fixed, stained with Crystal Violet and photographed in 10× magnification field. Invaded cells are indicated by black arrows in Y79 and WERI-Rb-1 cell controls. Control, scrambled and treated chambers of Y79 and WERI-Rb-1 are shown. (TIF) [file pone.0114800.s004.tif]

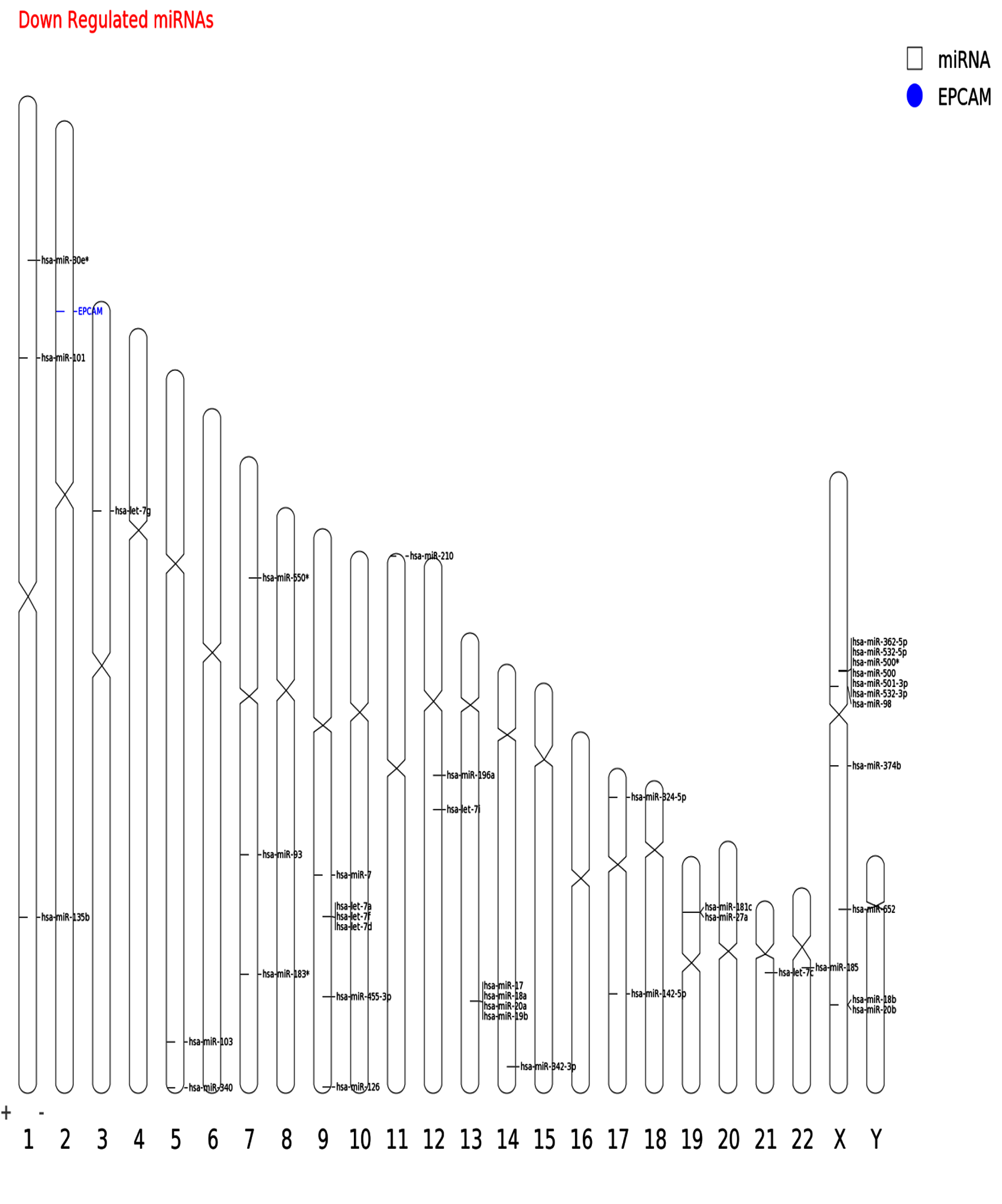

Supplement: S4 File — In silico representation of EpCAM downregulated miRNA on chromosomal regions. Chromosomal locations of significant down regulated miRNAs upon EpCAM silencing in Y79 cells. EpCAM is mapped to p-arm of Chromosome-2 (blue dot). miRNAs are labelled as lines on the 24 chromosomes. Polycistronic microRNAs-miR-17, miR-18a, miR-20a, miR-19b located on 13q31.3, miR-10, miR-30e located on chromosome-1 are associated with RB chromosomal gain regions. miRNAs (non-polycistronic), miR-362, miR-532, miR-500*, miR-500, miR-501*, miR-532* & miR-98 were located at Chromosomal-Xp11. (TIF) [file pone.0114800.s005.tif]

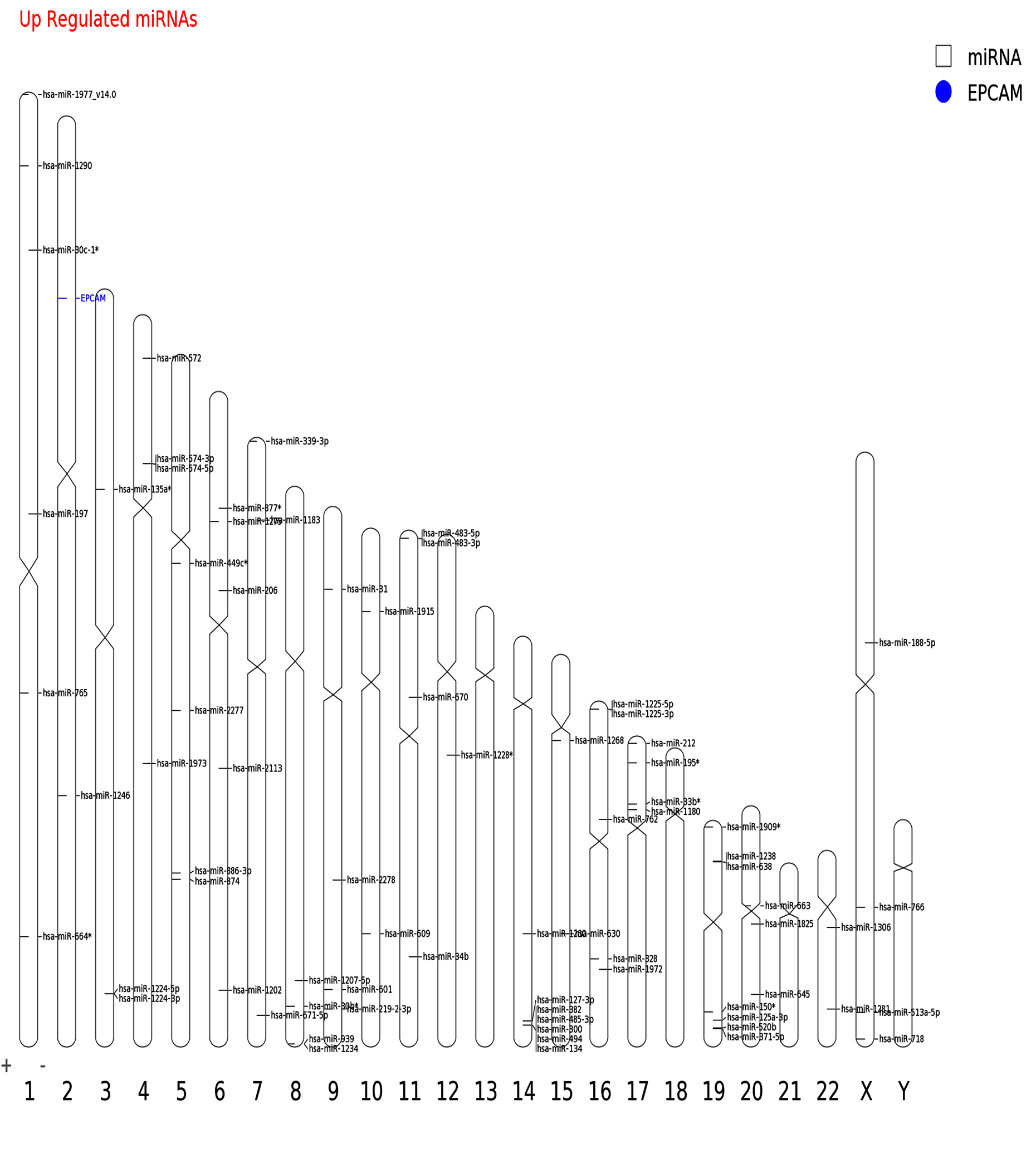

Supplement: S5 File — In silico representation of significantly up regulated miRNAs on EpCAM silencing in chromosomal regions. Details of chromosomal locations of significant miRNAs up regulated upon EpCAM silencing in Y79 cells. EpCAM is mapped to p-arm of Chromosome-2 (blue dot). miRNAs are labelled as lines on the 24 chromosomes. miR-127-3p, miR-382, miR-485, miR-300, miR-494, miR-134 map to chromosomal-14q32 region and miR-150*, miR-125a-3p, miR-520b, miR-371 map to chromosome-19q13.4 regions. (TIF) [file pone.0114800.s006.tif]
